# Multilocus Characterization Reveals Two Geographically Structured *Babesia* Lineages in Sheep from Southern and Southeastern Kazakhstan

**DOI:** 10.3390/pathogens15090984

**Published:** 2026-09-16

**Authors:** Assel Akhmetova, Alexander Ostrovskii, Anara Ryskeldina, Viktoriya Lutsay, Madina Kadyrova, Bolat Abdigulov, Rashid Karmaliyev, Alexandr Shevtsov

**Affiliations:** 1 Laboratory of Applied Genetics, National Center for Biotechnology, Astana 010000, Kazakhstan; 2 Institute of Veterinary Medicine and Agrotechnology, Zhangir Khan West Kazakhstan Agrarian Technical University, Uralsk 090009, Kazakhstan

**Keywords:** *Babesia*, sheep, Kazakhstan, *Babesia* sp. TRK, *B. motasi*, phylogenetic analysis, genetic diversity, molecular detection

## Abstract

In Kazakhstan, despite the diversity of ixodid ticks and extensive livestock movement throughout Central Asia, ovine *Babesia* infections are poorly characterized. The objective of this study was to determine the occurrence, genetic diversity and geographic distribution of *Babesia* spp. in sheep from southern and southeastern Kazakhstan. A total of 1458 blood samples from 51 settlements across five regions were screened by nested PCR targeting mitochondrial *cytb*. Positive samples were characterized by Sanger sequencing, with additional multilocus analysis based on 18S rRNA and mitochondrial *cox1* sequences. *Babesia* DNA was detected in 75/1458 samples (5.1%), with variation in PCR positivity among regions. Positive samples occurred in Turkestan (3.6%), Jambyl (7.2%), and Almaty (21.2%), but not in Kyzylorda or Jetisu. Phylogenetic analyses identified two geographically structured lineages. Nine Turkestan samples formed a lineage related to the *Babesia* sp. Xinjiang/Dunhuang group and were provisionally designated *Babesia* sp. TRK. The remaining 66 samples formed a *B. motasi* lineage comprising five *cytb* genotypes and showing closer affinity to the Lintan/Tianzhu group than to the Hebei/Ningxian group. Despite high 18S rRNA identity with *B. motasi*, mitochondrial markers revealed substantial divergence. The findings of the current study expand understanding of ovine *Babesia* diversity in Central Asia and emphasize the need for genomic characterization to resolve the taxonomic status of the detected lineages.

## 1. Introduction

Babesiosis is a tick-borne disease caused by intraerythrocytic protozoan parasites of the genus *Babesia* [1]. In sheep, *Babesia* infections may range from asymptomatic or persistent infections to acute hemolytic disease characterized by fever, anemia, jaundice, hemoglobinuria, and, in severe cases, death [2,3]. Several *Babesia* taxa infecting small ruminants have been characterized, including *Babesia ovis*, *B. motasi*, and *B. crassa*, while additional genetically distinct lineages have been described, particularly in Asia [4,5,6]. Among these parasites, *B. ovis* is regarded as one of the most pathogenic species in susceptible sheep and is primarily associated with transmission by *Rhipicephalus bursa* [2,3].

Considerable genetic and biological diversity has been reported among large ovine *Babesia* parasites [4,6]. Molecular studies in China have identified several *B. motasi*-like groups, including the Lintan/Tianzhu and Ningxian/Hebei lineages, which show substantial genetic differentiation and have subsequently been designated as *B. motasi lintanensis* and *B. motasi hebeiensis*, respectively [6,7]. DNA of *B. motasi*-like parasites has been detected in several ixodid tick species, including *Haemaphysalis longicornis* and *H. qinghaiensis*, although molecular detection in field-collected ticks alone does not establish vector competence. A genetically distinct ovine parasite, *Babesia* sp. Xinjiang, has also been described in China [4,5].

*Hyalomma anatolicum* is a recognized transmitting vector of this parasite, while molecular detection in *H. qinghaiensis* and *H. longicornis* suggests that its associations with ixodid ticks may be broader than currently established [5]. Together, these findings indicate considerable genetic diversity and unresolved taxonomic and vector relationships among ovine *Babesia* circulating in Central and East Asia. The variability of clinical presentation, together with the morphological similarity of piroplasms and the substantial species and genetic diversity within ovine *Babesia*, complicates reliable identification of the infecting taxon and accurate assessment of infection status at both the individual-animal and flock levels [4].

This is particularly relevant in apparently healthy or persistently infected animals, in which low parasitemia may obscure infection and limit the diagnostic value of clinical examination and conventional microscopy [8,9]. Laboratory diagnosis of ovine *Babesia* infection traditionally relies on microscopic examination of Giemsa-stained blood smears and serological assays, whereas molecular methods provide greater sensitivity for detecting low-parasitemia and persistent infections [10,11]. However, the taxonomic resolution provided by PCR depends strongly on the diagnostic strategy and the molecular target used [12,13]. Species-specific assays provide sensitive detection of predefined *Babesia* taxa but are inherently restricted to the species or genetic variants recognized by the selected primers or probes and may therefore fail to detect unexpected or divergent lineages [13]. In contrast, broad-range PCR assays targeting relatively conserved loci, followed by amplicon sequencing, permit the detection of a broader spectrum of *Babesia* taxa and can reveal previously unrecognized species or genotypes [12,13]. Nevertheless, highly conserved markers, particularly fragments of the 18S rRNA gene, may provide insufficient resolution for discriminating closely related *Babesia* species or genetic lineages [14,15]. More variable mitochondrial markers, such as *cytb* and *cox1*, can provide greater resolution among closely related lineages, while the combined analysis of nuclear and mitochondrial loci offers a more robust framework for characterizing genetically divergent *Babesia* taxa [15,16].

Kazakhstan has a diverse ixodid tick fauna, and field surveys in southern and southeastern regions have documented species belonging to the genera *Hyalomma*, *Rhipicephalus*, *Haemaphysalis*, and *Dermacentor* [17,18]. Molecular surveys have additionally detected several *Babesia* and *Theileria* species in ticks collected from livestock in Kazakhstan [18,19,20]. Recent molecular studies have also expanded knowledge of *Babesia* diversity in cattle and in ticks parasitizing livestock, whereas corresponding species-resolved data for *Babesia* infecting sheep in Kazakhstan remain scarce [20,21,22].

Consequently, the species composition, genetic diversity, and geographic distribution of *Babesia* lineages infecting sheep in Kazakhstan remain poorly characterized. Therefore, this study aimed to determine the occurrence and molecular diversity of *Babesia* spp. in adult sheep without reported clinical signs from southern and southeastern Kazakhstan using PCR, Sanger sequencing, and phylogenetic analysis of *cytb*, 18S rRNA, and *cox1* markers.

## 2. Materials and Methods

### 2.1. Ethical Approval

Ethical approval was obtained from the Ethics Committee of the National Center for Biotechnology for primary blood sample collection (Protocol No. 2, 4 April 2022), and subsequent molecular analysis of the archived samples for *Babesia* spp. (Protocol No. 4, 24 October 2023). Consent was obtained from the animal owners.

### 2.2. Study Design

This study was designed as a multi-regional cross-sectional molecular survey to assess the occurrence and species composition of *Babesia* spp. in adult sheep from southern and southeastern Kazakhstan. Animals were sampled from flocks without prior selection or stratification based on clinical status or suspected babesiosis, and no randomization was performed. Sampling was conducted during July–August 2022 and 2023 and included 51 settlements across five administrative regions: Kyzylorda, Turkestan, Jambyl, Almaty, and Jetisu. A total of 950 sheep were sampled in 2022 and 508 in 2023. The sampled settlements did not overlap between the two years. No formal a priori sample-size calculation was performed because the study was based on an available multi-regional convenience sample collected within an ongoing surveillance program.

### 2.3. Samples Collection

Blood samples were collected from adult ewes aged ≥3 years from privately owned farms in southern and southeastern Kazakhstan during July–August 2022 and 2023. A total of 1458 animals were sampled, with one blood sample obtained from each animal. Age was determined according to information provided by the animal owners. Approximately 10% of the animals available in each flock were selected by convenience during routine flock gathering. The sampled animals had no reported clinical signs at the time of sampling; however, no standardized clinical examination for babesiosis was performed at the time of sampling, and clinical status was not used as an inclusion criterion. Tick infestation was not systematically assessed.

Blood was collected into vacuum tubes containing dipotassium EDTA. Samples were maintained under refrigerated conditions (4–8 °C) during transportation to the laboratory and delivered within 48 h of collection. Upon arrival, the blood samples were processed for subsequent DNA extraction.

### 2.4. DNA Isolation

Genomic DNA was extracted from 300 µL of whole blood using a previously described silica-based method [23]. Briefly, erythrocytes were lysed with 900 µL of buffer containing 150 mM NH_4_Cl, 10 mM NaHCO_3_, and 1 mM EDTA, followed by centrifugation at 12,200× *g* for 5 min. The resulting cell pellet was resuspended in 100 µL of buffer containing 400 mM NaCl, 10 mM Tris-HCl (pH 8.0), and 2 mM EDTA and subjected to protein digestion with SDS (Sigma-Aldrich, Darmstadt, Germany) and Proteinase K (Magen, Guangzhou, China) at 50 °C for 2 h.

Nucleic acids were subsequently released in a guanidinium thiocyanate-based lysis solution containing 3.2 M GuSCN and adsorbed onto silicon dioxide particles (0.5–10 µm; Sigma-Aldrich, St. Louis, MO, USA; S5631) prepared according to Boom et al. [24]. Following sequential washing with guanidine-containing buffer and 75% ethanol, the sorbent was dried, and DNA was eluted in 100 µL of TE buffer (pH 8.0). DNA concentration was determined using a NanoDrop One spectrophotometer (Thermo Fisher Scientific, Madison, WI, USA).

### 2.5. PCR Detection and Multilocus Characterization of Babesia spp.

All 1458 DNA samples were screened for *Babesia* spp. using nested PCR targeting a fragment of the mitochondrial cytochrome b (*cytb*) gene. The *cytb* primers were previously developed by our group based on conserved regions of *Babesia* taxa, including *B. ovis*, *B. motasi*, *Babesia* sp. Xinjiang, and *Babesia* sp. Dunhuang (Supplementary Figure S1). Primer sequences and amplification conditions were described previously [22].

All *cytb*-positive samples were subjected to Sanger sequencing. For multilocus characterization, all nine samples assigned to the *Babesia* sp. TRK lineage were additionally analyzed using the 18S rRNA and mitochondrial cytochrome c oxidase subunit I (*cox1*) genes. At least 30% of samples assigned to the *B. motasi* lineage were selected by stratified random sampling, with settlement (geographic origin) and *cytb* genotype used as strata. This ensured representation of all sampled settlements and all detected *cytb* genotypes. Of the 66 *cytb*-positive samples assigned to the *B. motasi* lineage, 23 samples (34.8%) were selected for multilocus characterization, and all 23 yielded usable 18S rRNA and *cox1* sequences. Only unique genotypes were included in subsequent phylogenetic analyses.

The *cox1* fragment was amplified using the previously described primer system combining two forward primers, *Babesia*_*Cox1*_475-f1 and *Babesia*_*Cox1*_475-f2, in a single reaction [22]. Primers targeting the 18S rRNA gene were adopted from Heidarpour Bami et al. [25] and Hilpertshauser et al. [26].

PCR reactions were performed in a final volume of 25 µL using BioMaster HS-Taq PCR-Special (2×) Master Mix (cat. no. MH011-1020; Biolabmix, Novosibirsk, Russia) and 400 nM of each primer. First-round reactions contained 5 µL of genomic DNA, whereas nested reactions contained 2–3 µL of the first-round PCR product. Amplification was performed using a Mastercycler X50 thermal cycler (Eppendorf SE, Hamburg, Germany) under the locus-specific conditions described in the corresponding references. Each PCR run included positive and no-template negative controls. Amplicons of the expected size were visualized by agarose gel electrophoresis and subjected to Sanger sequencing.

### 2.6. DNA Sequencing and Phylogenetic Analysis

PCR amplicons were sequenced by the Sanger method using the BigDye Terminator v3.1 Cycle Sequencing Kit (Thermo Fisher Scientific, Vilnius, Lithuania) on an Applied Biosystems 3730xl DNA Analyzer (Applied Biosystems, Waltham, MA, USA). Chromatograms were inspected and edited, and consensus sequences were assembled using SeqMan v6.1 (DNASTAR, Madison, WI, USA) [27].

Initial sequence similarity searches were performed using BLASTn against the GenBank database (National Center for Biotechnology Information, NCBI). Reference sequences representing closely related and taxonomically relevant *Babesia* taxa were retrieved from GenBank for phylogenetic analysis. Separate multiple sequence alignments for *cytb*, 18S rRNA, and *cox1* were generated using ClustalW implemented in MEGA v12.1.2 [28]. Pairwise nucleotide identity and divergence between the obtained genotypes and selected reference sequences were calculated in MEGA v12.1.2 using the corresponding locus-specific alignments. To further assess the genetic structuring of the *cox1* lineages, Automatic Barcode Gap Discovery (ABGD) analysis was performed using the web-based SpartExplorer platform (Muséum national d’Histoire naturelle, Paris, France; last updated 20 June 2024) [29,30]. The analysis was conducted using the aligned *cox1* sequences. Pairwise genetic distances were calculated using the Kimura two-parameter (K80) model with a transition/transversion ratio of 2. The ABGD parameters were set as follows: relative gap width (X) = 1.5, minimum and maximum prior intraspecific divergence (Pmin and Pmax) = 0.001 and 0.1, respectively, 10 steps across the *p* range, and 20 bins. Both initial and recursive partitions were evaluated. The resulting partitions and their stability across the tested *p* values were recorded for comparison among the *cox1* lineages.

Phylogenetic trees were reconstructed using the maximum likelihood method in MEGA v12.1.2. The best-fit nucleotide substitution model was selected separately for each locus. The Kimura two-parameter model with gamma-distributed rate variation and invariant sites (K2 + G + I) was applied to the 18S rRNA alignment, whereas the General Time Reversible model with gamma-distributed rate variation and invariant sites (GTR + G + I) was used for the *cytb* and *cox1* alignments. Branch support was assessed using the standard nonparametric bootstrap method with 1000 replicates. Appropriate outgroup taxa were included to root the phylogenetic trees: *Theileria* spp. and *Cytauxzoon* spp. for the *cox1* analysis, and *Theileria* spp., *Cytauxzoon* spp., and *Toxoplasma gondii* for the 18S rRNA analysis. The novel sequences generated in this study were deposited in GenBank under accession numbers PZ803617–PZ803626 and PZ804642–PZ804643 (Supplementary Table S2).

### 2.7. Statistical Methods

PCR positivity was calculated as the proportion of *Babesia*-positive samples among all samples tested, with exact 95% confidence intervals. Because approximately 10% of animals per flock were sampled, and infection status may be correlated among animals within the same flock, we accounted for this clustering using a mixed-effects logistic regression model with Region as a fixed effect and flock/settlement identity as a random intercept, fitted to flock-level positive/total counts (binomial family, logit link; *lme4* package in R software v4.5.2) [31]. The overall effect of Region was assessed using a likelihood ratio test comparing the full model to a random-intercept-only null model. The intraclass correlation coefficient (ICC) was calculated to quantify the proportion of variance in PCR positivity attributable to flock-level clustering.

## 3. Results

### 3.1. Detection and Molecular Characterization of Babesia spp.

*Babesia* spp. DNA was detected by nested *cytb* PCR in 75 of 1458 sheep blood samples (5.1% of the sampled animals). Among the sampled animals, positive samples were identified in three of the five surveyed regions: Turkestan (9/251; 3.6%), Jambyl (22/304; 7.2%), and Almaty (44/208; 21.2%), whereas no positive samples were detected in Kyzylorda or Jetisu.

**Figure 1:**
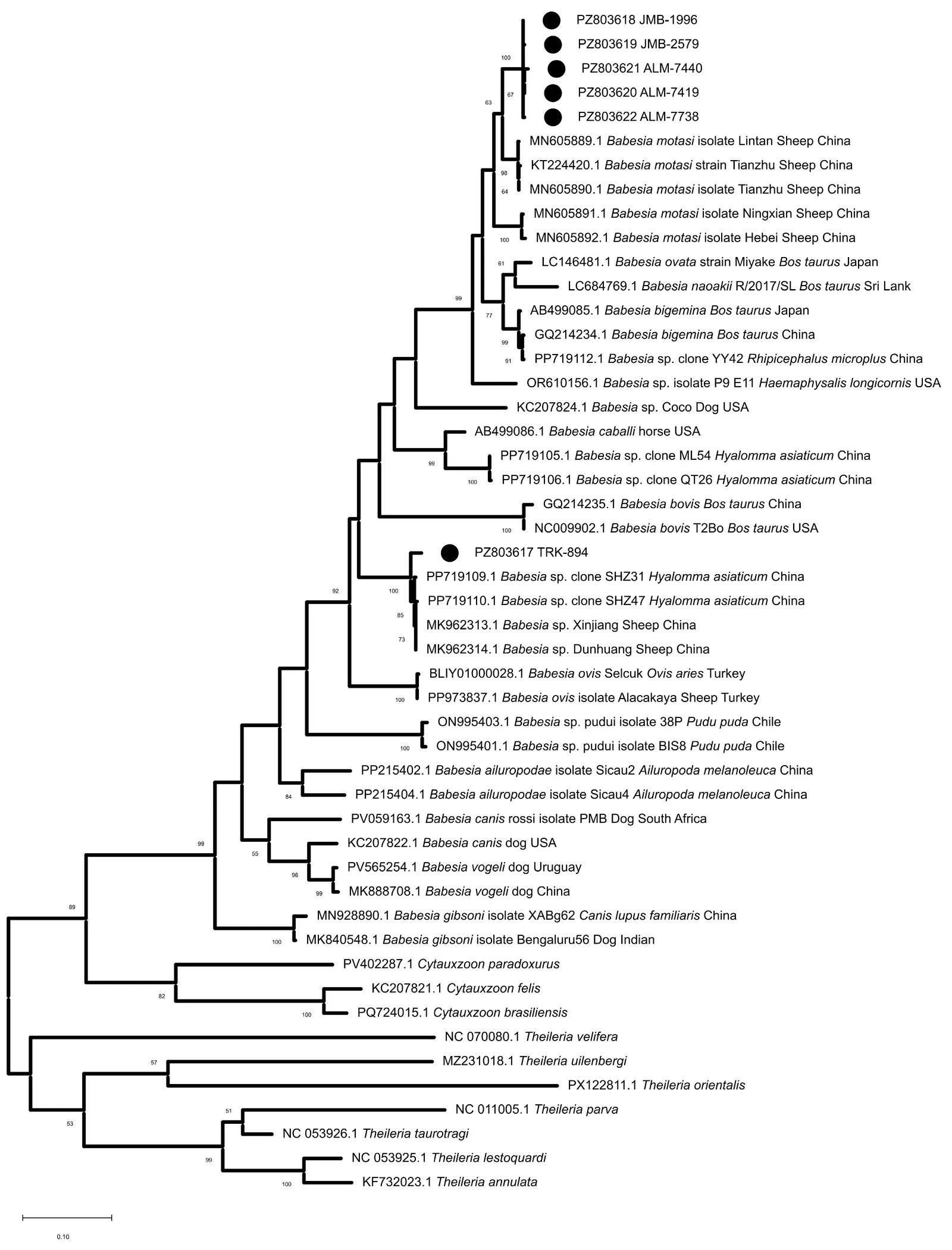
Maximum-likelihood phylogenetic tree of *Babesia* spp. based on a 499-bp fragment of the mitochondrial *cytb* gene. Phylogenetic reconstruction was performed in MEGA v12.1.2 using the GTR + G + I substitution model. Node labels indicate bootstrap support values (%), and the scale bar represents nucleotide substitutions per site. Sequences obtained in this study are indicated by black circles (●); reference sequences are labelled with their GenBank accession numbers.

Sanger sequencing of the *cytb* amplicons revealed two genetically distinct lineages (Figure 1). Nine sequences obtained from sheep in the Turkestan region formed a cluster with *Babesia* sp. Xinjiang, *Babesia* sp. Dunhuang, and related sequences previously reported from *Hyalomma asiaticum* ticks in China. The Kazakh sequences showed 98.4% nucleotide identity with *Babesia* sp. Xinjiang (MK962313.1), *Babesia* sp. Dunhuang (MK962314.1), and clone SHZ31 (PP719109.1) (Supplementary Figure S2). This lineage was provisionally designated *Babesia* sp. TRK.

The remaining 66 *cytb* sequences were assigned to *B. motasi* and comprised five closely related genotypes forming a distinct mitochondrial lineage within the *B. motasi* clade. These sequences showed 95.0–95.6% nucleotide identity with *B. motasi* reference sequences (Supplementary Figure S3) and formed a distinct branch most closely related to the Tianzhu and Lintan isolates from China (KT224420.1, MN605890.1, and MN605889.1) (Figure 1).

**Figure 2:**
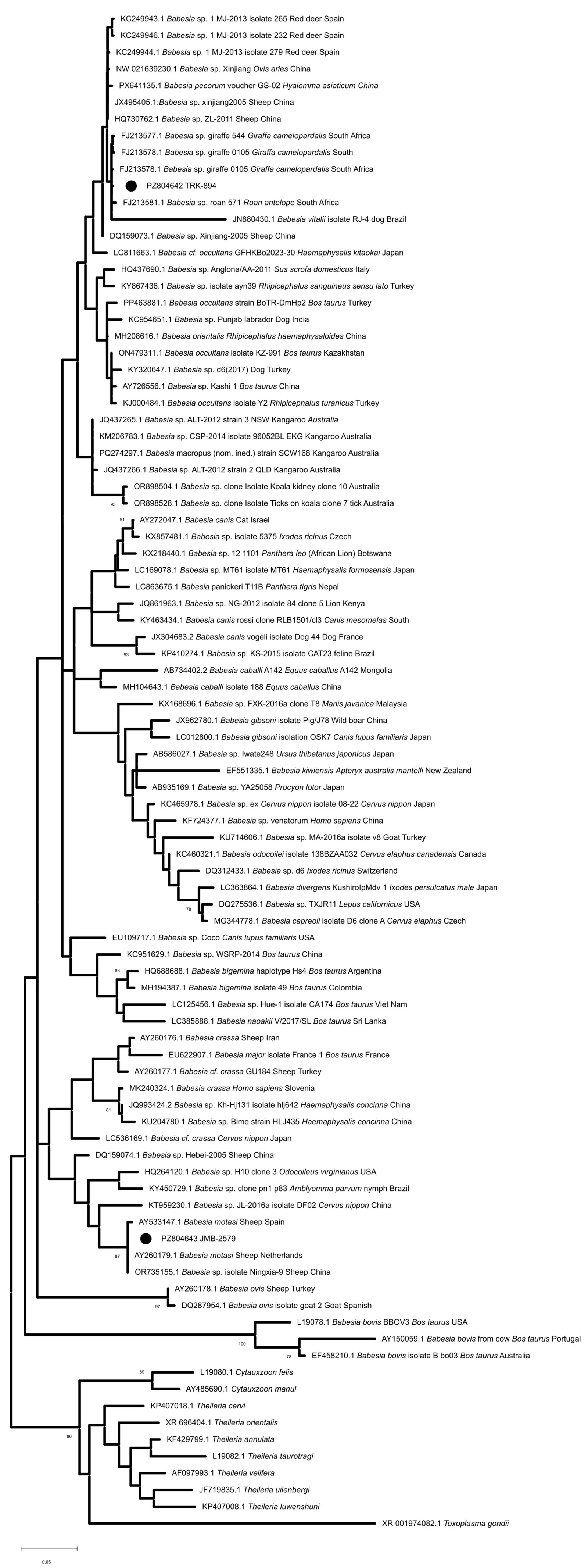
Maximum-likelihood phylogenetic tree of *Babesia* spp. based on a 378-bp fragment of the 18S rRNA gene. Phylogenetic reconstruction was performed in MEGA v12.1.2 using the K2 + G + I substitution model. Node labels indicate bootstrap support values (%), and the scale bar represents nucleotide substitutions per site. Sequences obtained in this study are indicated by black circles (●); reference sequences are labelled with their GenBank accession numbers.

Multilocus analysis supported the separation of the two lineages. Based on the 18S rRNA gene, *Babesia* sp. TRK showed 98.9% nucleotide identity with *Babesia* sp. Xinjiang (NW_021639230.1) and *Babesia* sp. giraffe (FJ213578.1) and clustered with these and related sequences in the phylogenetic tree (Figure 2). The 18S rRNA sequence representing the second lineage showed 100% nucleotide identity with *B. motasi* (AY260179.1) and *B. motasi* isolate Ningxian (OR735155.1) and clustered within the *B. motasi* clade, with 87% bootstrap support (Figure 2). These results support assignment of the Kazakh lineage to *B. motasi* at the species level.

**Figure 3:**
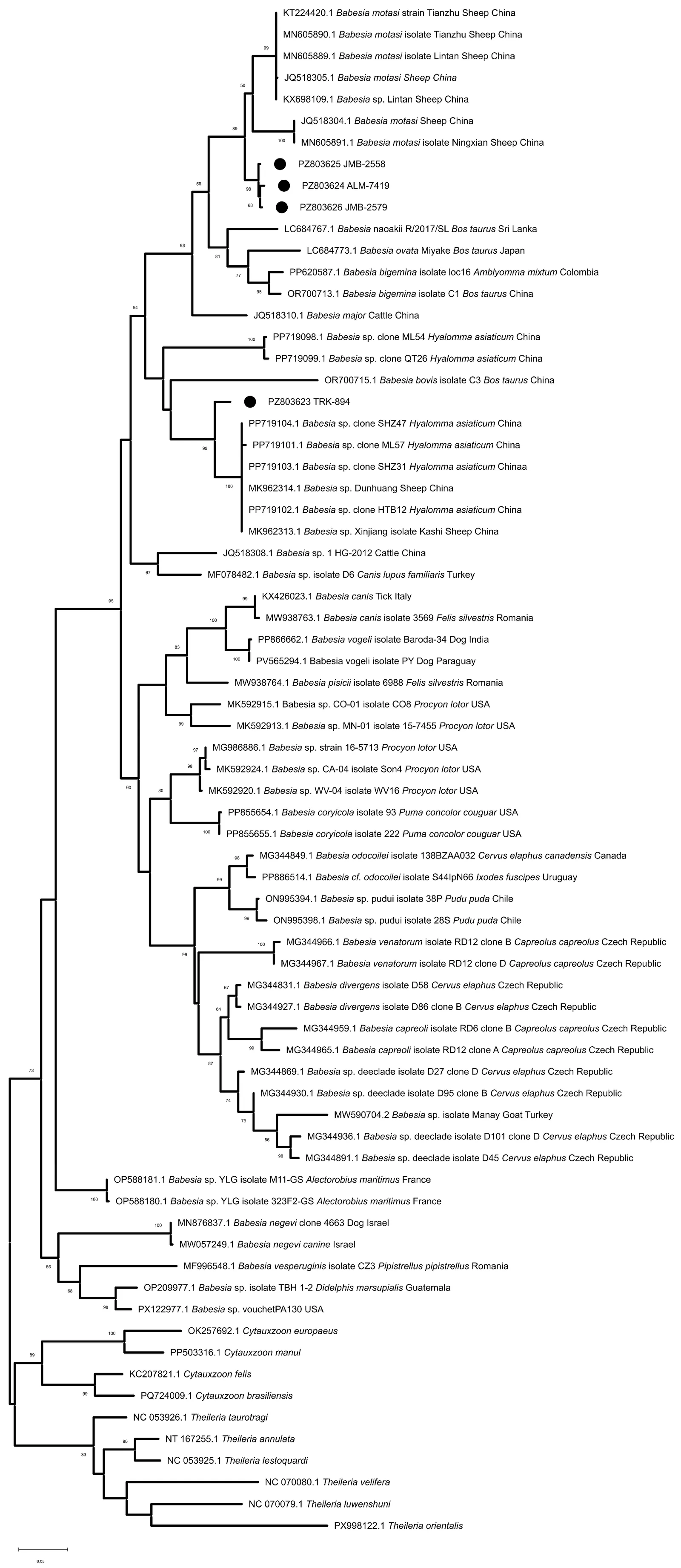
Maximum-likelihood phylogenetic tree of *Babesia* spp. based on a 503-bp fragment of the mitochondrial *cox1* gene. Phylogenetic reconstruction was performed in MEGA v12.1.2 using the GTR + G + I substitution model. Node labels indicate bootstrap support values (%), and the scale bar represents nucleotide substitutions per site. Sequences obtained in this study are indicated by black circles (●); reference sequences are labelled with their GenBank accession numbers.

Analysis of the *cox1* sequences showed the same separation. *Babesia* sp. TRK formed a distinct branch within the Xinjiang/Dunhuang-associated clade and showed 95.0–95.2% nucleotide identity with *Babesia* sp. Xinjiang (MK962313.1), *Babesia* sp. Dunhuang (MK962314.1), and related sequences from *H. asiaticum* ticks (PP719101–PP719104) (Figure 3).

**Figure 4:**
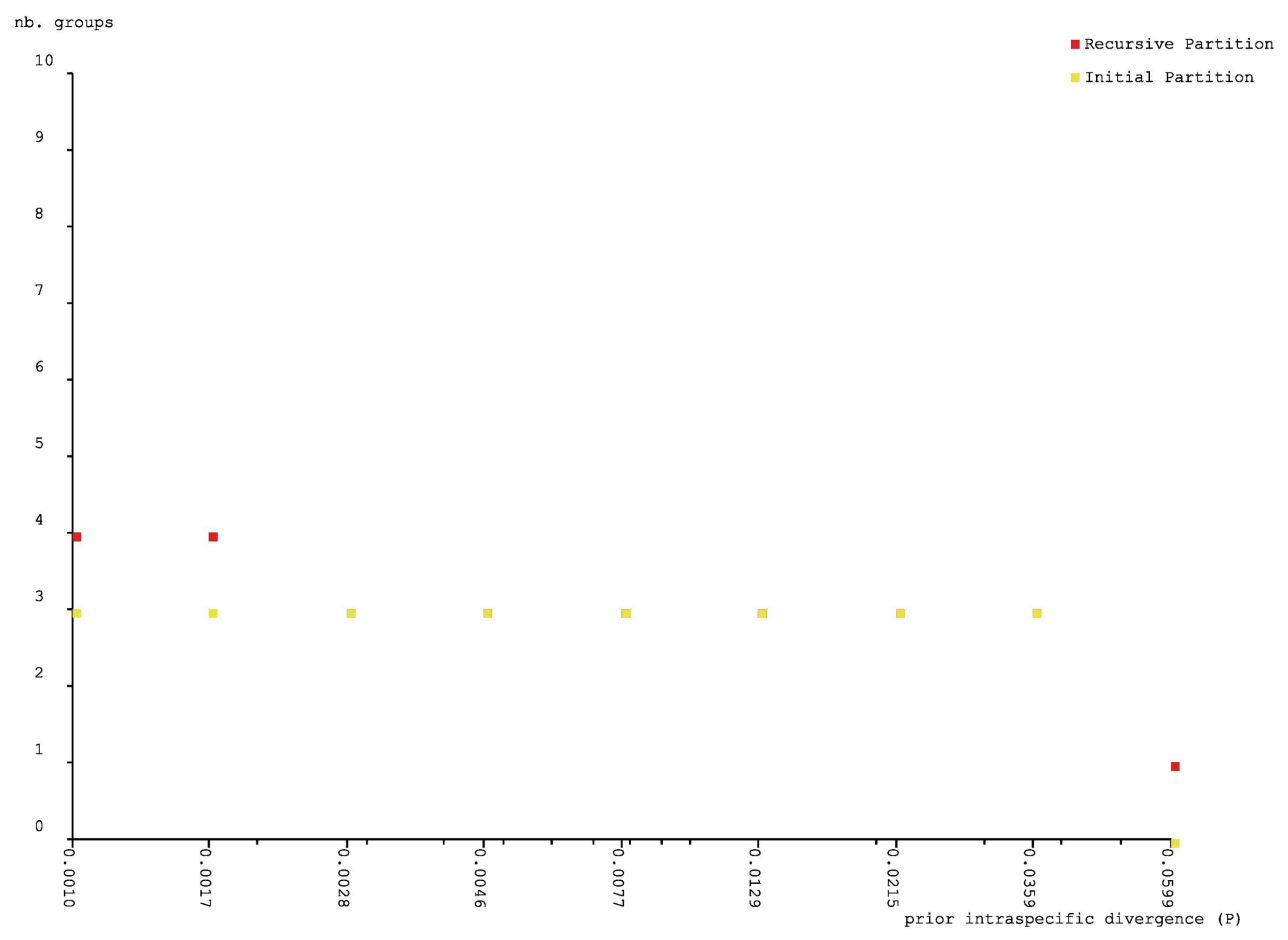
Automatic Barcode Gap Discovery analysis based on *cox1* sequences. The number of initial and recursive partitions is shown across the tested values of prior maximum intraspecific divergence (*p*). Three partitions were consistently recovered at *p* values ranging from 0.002783 to 0.035938 (0.28–3.59%), whereas all sequences were assigned to a single partition at *p* = 0.059948 (5.99%).

The second lineage comprised three *cox1* genotypes that showed 94.2–94.6% nucleotide identity (corresponding to approximately 5.4–5.8% p-distance) to the *B. motasi* Tianzhu and Lintan isolates (MN605890.1 and MN605889.1). Although these sequences clustered within the broader *B. motasi* clade, they formed a distinct mitochondrial lineage positioned phylogenetically between the Tianzhu/Lintan and Ningxian/Hebei sublineages. To further assess the genetic structuring of this lineage, we performed an Automatic Barcode Gap Discovery analysis. ABGD consistently recovered three partitions corresponding to the Kazakh, Ningxian/Hebei-associated, and Tianzhu/Lintan-associated lineages across a broad range of prior maximum intraspecific divergence values (*p* = 0.0028–0.0359; 0.28–3.59%) (Figure 4; Supplementary Table S3). At *p* = 0.0599 (5.99%), all sequences were assigned to a single partition. Thus, the approximately 5.4–5.8% *cox1* divergence observed between the Kazakh and Tianzhu/Lintan lineages was consistent with the partitioning pattern detected by ABGD and supported recognition of the Kazakh sequences as a distinct mitochondrial lineage within *B. motasi*.

### 3.2. Regional Occurrence of Detected Babesia Lineages

**Figure 5:**
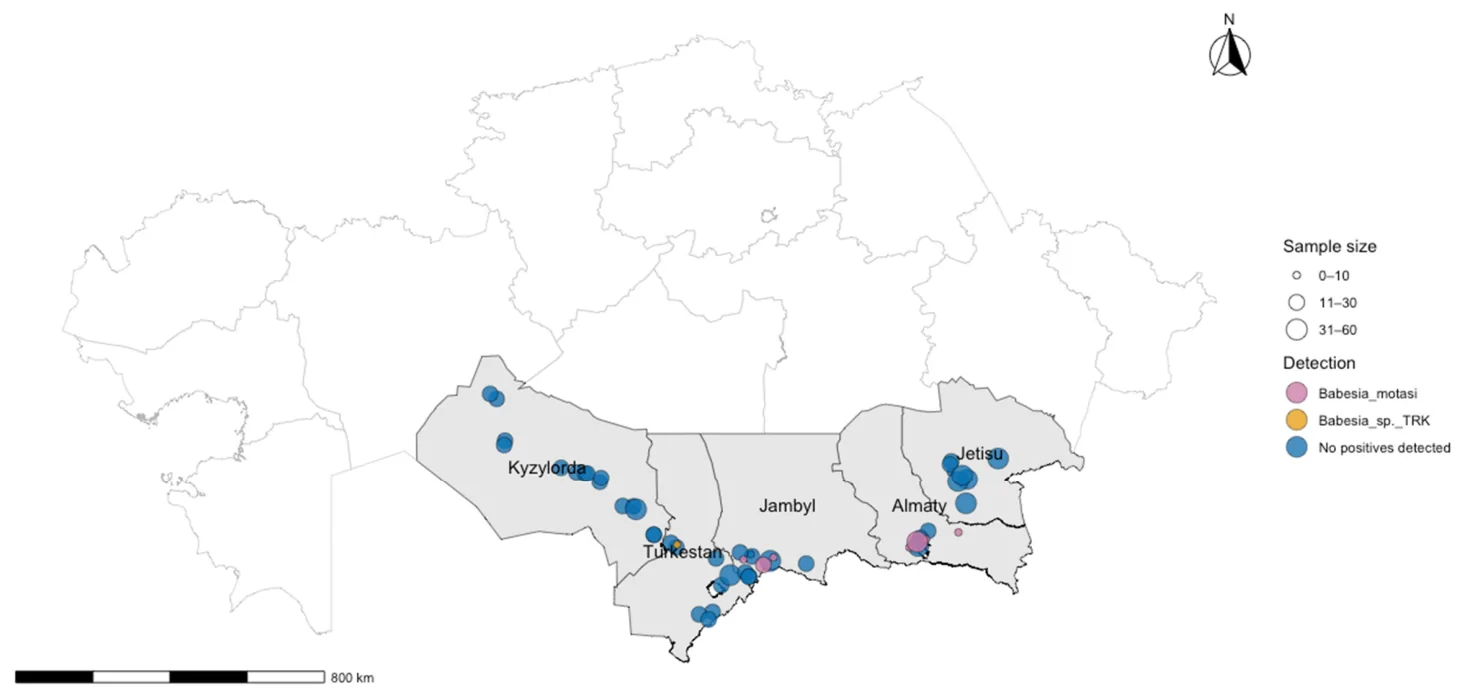
Map of Kazakhstan showing the five regions sampled in this study: Kyzylorda, Turkestan, Jambyl, Almaty, and Jetisu. Circle size is proportional to the number of samples collected at each location. Blue circles indicate PCR-negative samples, pink circles indicate the *B. motasi* lineage, and orange circles indicate *Babesia* sp. TRK.

*Babesia* sp. TRK was detected among sampled sheep in the Turkestan region (*n* = 9), with all positive samples originating from a single settlement, whereas *Babesia motasi* was detected in the Jambyl (*n* = 22) and Almaty (*n* = 44) regions (Figure 5, Table 1). Among the sampled animals, the highest proportion of *B. motasi*-positive samples was observed in the Almaty region.

The regional occurrence of *Babesia* lineages is presented in Figure 4 and Table 1. Detailed information on the sampled regions and locations is provided in Supplementary Table S1.

### 3.3. Statistical Analysis

PCR positivity varied across 51 settlements sampled, ranging from 0% to 60% (Supplementary Table S1). The intraclass correlation coefficient at the flock level was 0.76, indicating that most of the variability in PCR positivity was attributable to differences between settlements rather than between regions. After accounting for this clustering in a mixed-effects logistic regression, the overall effect of region (five regions) on PCR positivity was not statistically significant (likelihood ratio test: χ^2^ = 6.28, df = 4, *p* = 0.179).

## 4. Discussion

This multi-regional molecular survey provides new data on the occurrence and genetic diversity of *Babesia* spp. in sheep from southern and southeastern Kazakhstan. *Babesia* DNA was detected in three of the five surveyed regions, with the highest PCR positivity observed in Almaty and no positive samples detected in Kyzylorda or Jetisu. *Babesia* DNA was detected in 5.1% of the sampled sheep, with variation in observed PCR positivity among the surveyed regions and two distinct lineages identified: *B. motasi* and *Babesia* sp. TRK.

Phylogenetic analyses consistently placed *Babesia* sp. TRK within the Xinjiang/Dunhuang-associated lineage across all three *Babesia* molecular markers examined. The Kazakh lineage showed 98.4% nucleotide identity with *Babesia* sp. Xinjiang and *Babesia* sp. Dunhuang at *cytb* and 95.0–95.2% identity at *cox1*, whereas the more conserved 18S rRNA marker showed approximately 98.9% identity with closely related *Babesia* sequences. *Babesia* sp. Xinjiang was originally characterized as a genetically and biologically distinct ovine *Babesia* from China based on molecular, morphological, pathogenicity, and host/vector-related characteristics [32]. Subsequent molecular surveys demonstrated its occurrence in sheep and goats from several regions of China and confirmed *Hyalomma anatolicum* as its recognized transmitting vector, while parasite DNA was also detected in additional ixodid tick species [5]. Mitochondrial genome analysis further demonstrated that *Babesia* sp. Xinjiang and *Babesia* sp. Dunhuang form a distinct XJ/DH clade separated from the *B. motasi* lineages [33]. The consistent placement of the Kazakh sequences within this group, together with their mitochondrial divergence from available reference sequences, supports their close relationship to the Xinjiang/Dunhuang lineage but does not establish species-level identity. Therefore, the provisional designation *Babesia* sp. TRK is retained pending more extensive genomic and biological characterization.

The second lineage detected in Kazakhstan was phylogenetically associated with the *B. motasi* complex but showed a distinct multilocus sequence pattern. Previous studies of Chinese *B. motasi* have demonstrated the separation of the Lintan/Tianzhu and Hebei/Ningxian groups, with mitochondrial genome analyses placing these isolates in two distinct clades and subsequent whole-genome comparisons recognizing them as *B. motasi lintanensis* and *B. motasi hebeiensis*, respectively [6,33]. The Kazakh sequences were consistently more closely related to the Lintan/Tianzhu group than to the Hebei/Ningxian group. The 18S rRNA sequence showed 99.7–100.0% nucleotide identity with *B. motasi* reference sequences, whereas substantially greater differentiation was observed for the mitochondrial markers: 95.0–95.8% identity with the Lintan/Tianzhu group for *cytb* and 94.2–94.6% for *cox1*. The corresponding identities with the Hebei/Ningxian group were lower, reaching 92.2–92.8% for *cytb* and approximately 92.0–92.6% for *cox1*. The higher discriminatory resolution of mitochondrial *cox1* compared with the more conserved 18S rRNA marker has also been demonstrated in phylogenetic studies of *Babesia* spp. [15]. Thus, the multilocus data place the Kazakh lineage within the *B. motasi* complex and indicate a closer relationship to *B. motasi lintanensis*, while its substantial mitochondrial divergence argues against assigning it unequivocally to this subspecies on the basis of the partial loci analyzed here.

Molecular surveys of ovine *Babesia* have reported different parasite profiles in Iran, China, and neighboring regions; however, differences in study design, molecular targets, and diagnostic approaches make PCR detection rates difficult to compare directly [34,35,36]. In Iran, molecular surveys have consistently identified *B. ovis* as the principal ovine *Babesia* species, whereas *B. motasi* was absent or rarely detected in the investigated populations [35,36]. In contrast, molecular surveys in China have demonstrated widespread *B. motasi*-like infections and substantial genetic heterogeneity within this parasite complex [4]. Particularly relevant to Kazakhstan, sheep from the northwestern Chinese border region were PCR-positive for both *B. motasi*-like parasites (18.6%) and the *Babesia* Xinjiang-associated lineage (5.0%), including detections in Bole and Jimunai, which border Kazakhstan [34]. This geographic pattern is concordant with the phylogenetic affinities observed in the present study, although the available data are insufficient to infer direct cross-border transmission. Recent evidence further supports the possibility that sheep may harbor *Babesia* species traditionally associated with other livestock hosts. A recent molecular study detected *Babesia bovis* DNA in a sheep raised together with cattle, as well as in cattle and *Rhipicephalus microplus* ticks from the same farm, suggesting possible circulation of a common parasite lineage in a mixed cattle–sheep production system [37]. This finding broadens the recognized host range of *Babesia* spp. and highlights the potential importance of sheep in the epidemiology of *Babesia* infections.

*Babesia* DNA was detected in adult sheep without reported clinical signs, particularly in Almaty (21.2%). This finding may be compatible with subclinical or chronic infection, but this interpretation cannot be confirmed because standardized clinical and quantitative parasitaemia data were not available. In endemic settings, repeated exposure to tick-borne *Babesia* may result in partial protective immunity (premunition), which may contribute to the absence of clinical signs despite infection. This pattern may be compatible with enzootic stability, although the present study cannot establish this directly because longitudinal, quantitative parasitaemia, serological, and standardized clinical data were not available.

The absence of *B. ovis* from the present dataset contrasts with reports from Iran, where this species is consistently detected in sheep and is regarded as the principal *Babesia* species associated with ovine babesiosis [35,36]. The *cytb* primers used in the present study were designed from conserved regions represented across multiple ovine *Babesia* taxa, including *B. ovis* [22]. Nevertheless, the absence of *B. ovis* in the present dataset does not preclude its presence in Kazakhstan, as the study was based on a convenience sample from five regions and covered a limited seasonal period [18,38]. The seasonal dynamics of potential tick vectors may also be relevant to the interpretation of the absence of *B. ovis*. Recent entomological surveys in southern Kazakhstan have demonstrated pronounced seasonal variations in ixodid tick activity. *Hyalomma* spp. are among the predominant livestock-associated ticks in this region, although the timing of peak activity differs among species. In particular, *H. scupense* has been reported to reach peak activity in June, whereas *H. anatolicum* shows pronounced summer activity, with peak abundance in July and substantial activity persisting into August. In contrast, *H. asiaticum* is characterized predominantly by spring activity, with a peak in May and a marked decline during summer [17,39]. *Rhipicephalus bursa* has also been documented among ixodid ticks in southern Kazakhstan [40]. Thus, the July–August sampling period overlapped with the period of high activity of some potential vectors, particularly *H. anatolicum*, but may not have captured the peak activity of all vector species. Consequently, seasonal variation in vector abundance and transmission intensity, together with potentially low or transient parasitaemia, may have contributed to the failure to detect *B. ovis* in the present dataset.

The variation in observed PCR positivity among the surveyed regions may reflect differences in local transmission environments, although the underlying determinants cannot be resolved from our data. Previous field surveys in eastern and southern Kazakhstan have demonstrated a substantial spatial variation in ixodid tick communities and detected *Babesia* and *Theileria* DNA in field-collected ticks, with *Dermacentor marginatus* and *Hyalomma asiaticum* among the most abundant hard tick species recorded [18]. More recent surveys in the Almaty, Jambyl, and Turkestan regions have likewise documented marked regional diversity in livestock-associated ixodid tick communities [17]. These findings provide an ecological context for interpreting the regional occurrence of *Babesia* sp. TRK and the *B. motasi* lineage observed in the present study. However, associations between these lineages and specific tick vectors cannot be inferred from the present study. The observed geographic structure may be shaped by multiple interacting factors, including the composition and distribution of potential tick vectors, climatic conditions, grazing practices, and livestock movements, whose relative contributions were not assessed in the present study.

Several limitations should be considered when interpreting the present findings. The study was based on a multi-regional convenience sample without a formal a priori sample-size calculation, and sampling intensity differed among regions and between sampling years. Although all PCR-positive samples were characterized using *cytb*, 18S rRNA and *cox1* sequencing was performed only for a subset of samples. Animals were sampled irrespective of clinical status, and no standardized clinical examination for babesiosis was performed. Because individual sheep were sampled within flocks and settlements, observations may not have been statistically independent; therefore, regional differences in PCR positivity were assessed using mixed-effects logistic regression accounting for settlement-level clustering. Tick infestation was not systematically assessed, and ticks were not collected from the sampled sheep; therefore, associations between the detected *Babesia* lineages and particular potential vector species could not be evaluated. In addition, the survey covered only five regions and a limited seasonal sampling period. Therefore, the reported PCR positivity values should be interpreted as descriptive findings for the sampled animals rather than as population-level prevalence estimates and should not be extrapolated to the entire sheep population of Kazakhstan.

Further studies should extend molecular surveillance of ovine *Babesia* to additional regions of Kazakhstan and incorporate simultaneous sampling of sheep and associated ticks to clarify the geographic distribution and potential vector associations of the detected lineages. Previous studies have shown that combined molecular characterization of parasites from small ruminants and ixodid ticks is informative for resolving the epidemiology of *B. motasi*-like and *Babesia* sp. Xinjiang lineages [4,5]. In particular, complete mitochondrial and nuclear genomic characterization of *Babesia* sp. TRK and the *B. motasi*-associated lineage will be required to resolve their taxonomic relationships more precisely, as genome-scale comparisons have provided substantially greater resolution among closely related *B. motasi* lineages than individual molecular markers. [6]. Integration of molecular data with standardized clinical assessment would additionally help determine whether the genetically distinct lineages detected in Kazakhstan differ in their pathogenic potential.

## 5. Conclusions

This multi-regional molecular survey detected *Babesia* DNA in 5.1% of sheep sampled in southern and southeastern Kazakhstan and revealed substantial settlement-level heterogeneity in PCR positivity. Multilocus analysis identified two genetically distinct lineages. *Babesia* sp. TRK was restricted to the Turkestan region and was closely related to the Xinjiang/Dunhuang-associated group, while the second lineage, detected in Jambyl and Almaty, clustered within the *B. motasi* complex and was more closely related to *B. motasi lintanensis* than to *B. motasi hebeiensis*. However, substantial mitochondrial divergence from available reference sequences indicates that its precise taxonomic position requires further genomic characterization. These findings expand current knowledge of the genetic diversity and geographic structure of ovine *Babesia* in Central Asia.

## Figures and Tables

**Table 1:** Regional occurrence and PCR positivity of *Babesia* spp. detected in sheep blood samples from southern and southeastern Kazakhstan.

Region	Settlements, *n*	Positive/Tested, *n*	PCR-Positive Animals, *n* (%)	95% Exact CI	*Babesia* sp. TRK, *n*	*Babesia Motasi*, *n*
Kyzylorda	16	0/395	0%	0–0.93%	-	-
Turkestan	9	9/251	3.6%	1.65–6.70%	9	0
Jambyl	11	22/304	7.2%	4.59–10.75%	0	22
Almaty	7	44/208	21.2%	15.81–27.34%	0	44
Jetisu	8	0/300	0%	0–1.22%	-	-
Overall	51	75/1458	5.14%	4.07–6.41%	9	66

## Data Availability

The data produced within the current study are openly available in NCBI and GenBank at https://www.ncbi.nlm.nih.gov/nuccore (accessed on 28 August 2026), reference numbers PZ803617–PZ803626, PZ804642–PZ804643. R code for Figure 4 is available from https://github.com/asselakhmet/Spatial_analysis_Babesia_spp_Kazakhstan- (accessed on 10 August 2026).

## Supplementary Materials

The following supporting information can be downloaded at https://www.mdpi.com/article/10.3390/pathogens15090984/s1, Supplementary Table S1: Names of sampled settlements, geographic coordinates (from Google Maps v11.65.00), and distribution of *Babesia* spp. infection in adult ewes from 51 settlements across 5 provinces of Kazakhstan; Supplementary Table S2: GenBank accession numbers of *Babesia* sequences generated in this study, including sample identifiers and target genes; Supplementary Table S3: Automatic Barcode Gap Discovery (ABGD) analysis of *cox1* sequences within the *Babesia motasi* lineage. Supplementary Figure S1: Alignment of *cytb* primer-binding regions. Supplementary Figure S2: Pairwise sequence identity and divergence matrices of *Babesia* sp. TRK and closely related *Babesia* sequences based on *cytb*, 18S rRNA, and *cox1*; Supplementary Figure S3: Pairwise sequence identity and divergence matrices of *Babesia motasi* and closely related *Babesia* sequences based on *cytb*, 18S rRNA, and *cox1*.

## Abbreviations

The following abbreviations are used in this manuscript:

*cytb*	Cytochrome b gene
PCR	Polymerase chain reaction
nPCR	Nested polymerase chain reaction
18S rRNA	18S ribosomal RNA
*cox1*	Cytochrome c oxidase subunit I
