# Supplementary material for: Multilocus Characterization Reveals Two Geographically Structured Babesia Lineages in Sheep from Southern and Southeastern Kazakhstan
Source: Pathogens. 2026 Sep 16;15(9):984. doi: 10.3390/pathogens15090984 (PMC13610672; doi:10.3390/pathogens15090984)
Supplement: Supplementary file 1 [file pathogens-15-00984-s001.zip › Supplementary Table S1.pdf]

Akhmetova A., Ostrovskii A., Ryskeldina A., Lutsay V., Kadyrova M., Shevtsov A. (2026): Molecular detection and characterization of *Babesia* spp. in sheep blood samples collected from Southern Kazakhstan regions. – Supplementary Table S1

Supplementary Table S1. Names of sampled settlements, geographic coordinates (from Google Maps), distribution of *Babesia* spp. infection in adult ewes from 51 settlements across 5 provinces of Kazakhstan (sampling conducted in July–August 2022 and 2023)

**Province (oblast)**	**Administrative district**	**Settlement**	**Latitude**	**Longitude**	**N tested using PCR**	**N positive**		***Babesia motasi***		***Babesia* sp. TRK**	
						**N pos.**	**% pos.**	**N pos.**	**% pos.[a]**	**N pos.**	**% pos.[a]**
**Turkistan**	**Subtotal province**				**251**	**9**	**4**	**0**	**0**	**9**	**4**
	Sayram	Karasu	42.405962	69.795713	20	0	0				
	Ordabasy	Burdjar	43.296298,	68.207760	11	0	0				
	Tulkibas	Abay	42.641091	70.114274	47	0	0				
	Kazygurt	Sharbulak	41.774444	69.490278	30	0	0				
	Sauran	Karashyk	43.357736	68.231624	29	9	31				
		Chernak	43.399573	68.016379	29	0	0				
	Saryagash	Zhilga	41.718253	69.013000	27	0	0				
		Akzhar	41.598945	69.347344	28	0	0				
	Baydibek	Kenestobe	43.036235	69.610353	30	0	0				
**Jambyl**	**Subtotal province**				**304**	**22**	**7**	**22**	**7**	**0**	**0**
	Bayzak	Kokbastau	43.057866	71.643991	30	3	10				
		Bayzak	42.977381	71.529450	60	0	0				
	Jambyl	Ornek	43.083320	70.878926	30	0	0				
		Karatobe	42.923230	71.194118	23	12	52				
		Ernazar	43.132980	70.839940	10	0	0				
	Turar Ryskulova	Abay rural district	42.912313	72.800952	29	0	0				
	Talas	Berikkainar	43.012481	70.580326	30	7	23				
		Karatau	43.175515	70.459523	23	0	0				
	Zhualy	Shinbulak	42.706488	70.650511	29	0	0				
		Kolbastau	42.607500	70.784167	27	0	0				
		Aksay	42.604679	70.762693	13	0	0				
**Kyzylorda**	**Subtotal province**				**395**	**0**	**0**	**0**	**0**	**0**	**0**
	Karmakshy	3-d Internatsional	45.191667	64.128889	19	0	0				
	Kyzylorda	Kyzylorda	44.842544	65.502563	22	0	0				
		Kosshynyrau rural district	44.947222	65.544167	29	0	0				
	Aral	Zhaksykylysh	46.824752	61.854119	15	0	0				
		Akkum	46.942778	61.620000	14	0	0				
	Kazaly	Ayteke Bi	45.835934	62.148317	28	0	0				
		Sarykol rural district	45.732557	62.115942	29	0	0				
	Zhalagash	Zhalagash	45.080281	64.681333	30	0	0				
	Zhanakorgan	Akkorgan	43.592500	67.411111	28	0	0				
		Kirkense	43.592500	67.411111	27	0	0				
	Shieli	Telekol rural district	44.259722	66.685556	30	0	0				
		Zhuantobe	44.267588	66.311218	29	0	0				
		Shieli	44.178889	66.732778	5	0	0				
		Kerdeli	44.192500	66.781944	34	0	0				
	Syrdariya	Terenozek	45.051944	64.980556	29	0	0				
		Askar Tekmagambetov rural district	45.057148	65.052480	27	0	0				
**Almaty**	**Subtotal province**				**208**	**44**	**21**	**44**	**21**	**0**	**0**
	Enbekshikazakh	Karashota	43.649992	78.183065	20	2	10				
	Jambyl	Mynbaev	43.294611	76.450790	29	8	28				
	Ile	Zhetygen	43.681261	77.116046	20	0	0				
		Karaoy	43.517111	76.833212	20	0	0				
		Baiserke	43.489634	77.040615	19	1	5				
	Karasay	Karatobe	43.424313	76.731262	50	33	66				
		Koktogan	43.328223	76.764203	50	0	0				
**Jetisu**	**Subtotal province**				**300**	**0**	**0**	**0**	**0**	**0**	**0**
	Aksu	Esbolatov	45.412154	79.587680	50	0	0				
	Eskeldi	Karabulak	44.909355	78.489031	50	0	0				
	Kerbulak	Altynemel	44.333643	78.451634	50	0	0				
	Karatal	Kishitobe	45.345439	77.929291	20	0	0				
		Kalpe	45.090947	77.907255	10	0	0				
		Kokpekty	45.275010	77.884968	20	0	0				
	Koksu	Mukry	44.859765	78.171737	50	0	0				
	Taldykorgan	Utenay	45.006769	78.318657	50	0	0				
**Total**					**1458**	**75**	**5,1**	**66**	**4,5**	**9**	**0,6**

[a]Percentage by region
