# Supplementary material for: Multilocus Characterization Reveals Two Geographically Structured Babesia Lineages in Sheep from Southern and Southeastern Kazakhstan
Source: Pathogens. 2026 Sep 16;15(9):984. doi: 10.3390/pathogens15090984 (PMC13610672; doi:10.3390/pathogens15090984)
Supplement: Supplementary file 1 [file pathogens-15-00984-s001.zip › Supplementary Table S2.pdf]

Supplementary Table S2. GenBank accession numbers of *Babesia* sequences generated in this study, including sample identifiers and target genes.

Sample ID	Host species	Species	Gene	GenBank accession number
TRK-894	sheep	*Babesia* sp. TRK	*cytb*	PZ803617
JMB-1996	sheep	*Babesia motasi*	*cytb*	PZ803618
JMB-2579	sheep	*Babesia motasi*	*cytb*	PZ803619
ALM-7419	sheep	*Babesia motasi*	*cytb*	PZ803620
ALM-7440	sheep	*Babesia motasi*	*cytb*	PZ803621
ALM-7738	sheep	*Babesia motasi*	*cytb*	PZ803622
TRK-894	sheep	*Babesia* sp. TRK	*cox1*	PZ803623
ALM-7419	sheep	*Babesia motasi*	*cox1*	PZ803624
JMB-2558	sheep	*Babesia motasi*	*cox1*	PZ803625
JMB-2579	sheep	*Babesia motasi*	*cox1*	PZ803626
TRK-894	sheep	*Babesia* sp. TRK	18S rRNA	PZ804642
JMB-2579	sheep	*Babesia motasi*	18S rRNA	PZ804643

Abbreviations: ALM,Almaty Region; JMB, Jambyl Region; TRK, Turkestan Region; *cytb,* cytochromeb gene; 18S rRNA, 18S ribosomal RNA gene; *cox1,* cytochrome c oxidase subunit I gene.
