# Supplementary material for: Multilocus Characterization Reveals Two Geographically Structured Babesia Lineages in Sheep from Southern and Southeastern Kazakhstan
Source: Pathogens. 2026 Sep 16;15(9):984. doi: 10.3390/pathogens15090984 (PMC13610672; doi:10.3390/pathogens15090984)
Supplement: Supplementary file 1 [file pathogens-15-00984-s001.zip › Supplementary Table S3.pdf]

Akhmetova A., Ostrovskii A., Ryskeldina A., Lutsay V., Kadyrova M., Abdigulov B., Karmaliyev R., Shevtsov A. (2026): Multilocus Characterization Reveals Two Geographically Structured *Babesia* Lineages in Sheep from Southern and Southeastern Kazakhstan

Supplementary Table S3. Automatic Barcode Gap Discovery (ABGD) analysis of *cox1* sequences within the *Babesia motasi* lineage. ABGD consistently recovered three partitions over a broad range of prior maximum intraspecific divergence values (P = 0.002783–0.035938), corresponding to the Kazakh, Ningxian/Hebei-associated, and Tianzhu/Lintan-associated lineages. At P = 0.059948, all sequences were assigned to a single partition. Numerical partition identifiers correspond to those generated by SparteXplorer.

Sequence ID	Taxon / isolate	Host	Country	ABGD partition	Interpreted lineage
PZ803624	ALM-7419	Sheep	Kazakhstan	3	Kazakh lineage
PZ803625	JMB-2558	Sheep	Kazakhstan	3	Kazakh lineage
PZ803626	JMB-2579	Sheep	Kazakhstan	3	Kazakh lineage
MN605891.1	Babesia motasi isolate Ningxian	Sheep	China	2	Ningxian/Hebei-associated lineage
JQ518304.1	Babesia motasi	Sheep	China	2	Ningxian/Hebei-associated lineage
KT224420.1	Babesia motasi strain Tianzhu	Sheep	China	5	Tianzhu/Lintan-associated lineage
KX698109.1	Babesia sp. Lintan	Sheep	China	5	Tianzhu/Lintan-associated lineage
MN605890.1	Babesia motasi isolate Tianzhu	Sheep	China	5	Tianzhu/Lintan-associated lineage
MN605889.1	Babesia motasi isolate Lintan	Sheep	China	5	Tianzhu/Lintan-associated lineage
JQ518305.1	Babesia motasi	Sheep	China	5	Tianzhu/Lintan-associated lineage

ABGD parameters/results		
**Prior maximum intraspecific divergence (P)**	**Initial partition**	**Recursive partition**
0.001000	3	4
0.001668	3	4
0.002783	3	3
0.004642	3	3
0.007743	3	3
0.012915	3	3
0.021544	3	3
0.035938	3	3
0.059948	1	1
